# Current Management and Therapeutic Strategies for Cerebral Amyloid Angiopathy

**DOI:** 10.3390/ijms22083869

**Published:** 2021-04-08

**Authors:** Yasuteru Inoue, Yukio Ando, Yohei Misumi, Mitsuharu Ueda

**Affiliations:** 1 Department of Neurology, Graduate School of Medical Sciences, Kumamoto University, Kumamoto 860-8556, Japan; misumiyohei@hotmail.co.jp (Y.M.); mitt@rb3.so-net.ne.jp (M.U.); 2 Department of Amyloidosis Research, Nagasaki International University, Sasebo 859-3298, Japan; andoy709@kumamoto-u.ac.jp

**Keywords:** cerebral amyloid angiopathy, amyloid β, intracerebral hemorrhage, cerebral microbleeds, superficial siderosis, proteomic analyses

## Abstract

Cerebral amyloid angiopathy (CAA) is characterized by accumulation of amyloid β (Aβ) in walls of leptomeningeal vessels and cortical capillaries in the brain. The loss of integrity of these vessels caused by cerebrovascular Aβ deposits results in fragile vessels and lobar intracerebral hemorrhages. CAA also manifests with progressive cognitive impairment or transient focal neurological symptoms. Although development of therapeutics for CAA is urgently needed, the pathogenesis of CAA remains to be fully elucidated. In this review, we summarize the epidemiology, pathology, clinical and radiological features, and perspectives for future research directions in CAA therapeutics. Recent advances in mass spectrometric methodology combined with vascular isolation techniques have aided understanding of the cerebrovascular proteome. In this paper, we describe several potential key CAA-associated molecules that have been identified by proteomic analyses (apolipoprotein E, clusterin, SRPX1 (sushi repeat-containing protein X-linked 1), TIMP3 (tissue inhibitor of metalloproteinases 3), and HTRA1 (HtrA serine peptidase 1)), and their pivotal roles in Aβ cytotoxicity, Aβ fibril formation, and vessel wall remodeling. Understanding the interactions between cerebrovascular Aβ deposits and molecules that accumulate with Aβ may lead to discovery of effective CAA therapeutics and to the identification of biomarkers for early diagnosis.

## 1. Introduction

Sporadic cerebral amyloid angiopathy (CAA) is characterized by progressive accumulation of cerebrovascular amyloid β (Aβ) in the walls of leptomeningeal arteries and cortical capillaries in the brain. Loss of integrity of cerebral blood vessels caused by cerebrovascular Aβ deposits has led to spontaneous lobar intracerebral hemorrhage (ICH) and cognitive impairment [1]. Population-based postmortem studies indicated that the prevalence of CAA is 20–40% in elderly people without dementia and 50–60% in elderly people with dementia [2,3,4,5]. With regard to ICH, CAA-related ICH accounts for 20% of all types of ICH [6]. We previously investigated the locations of hematomas in very elderly patients (≥80 years old) with ICHs and compared them with those of patients with ICHs who were younger than 80 years. We found that hematomas often occurred in the subcortex in very elderly patients with ICH, and a high prevalence of CAA was strongly implicated in ICH occurrence [7]. Furthermore, a recent meta-analysis demonstrated that the annual recurrent ICH risk was higher in patients with CAA-related ICH compared with patients with CAA-unrelated ICH (7.4% vs. 1.1%) [8]. Developing a strategy to treat CAA is urgently needed, but the understanding of its pathogenesis and identification of molecules that may serve as therapeutic targets remain to be elucidated. This review aims to: summarize the clinical, pathological, and radiological features of CAA; describe current management strategies for CAA; discuss proposed mechanisms of formation of CAA; and clarify the CAA-related molecules that may be therapeutic targets. 

## 2. Neuropathology of CAA-Affected Vessels

**Figure 1:**
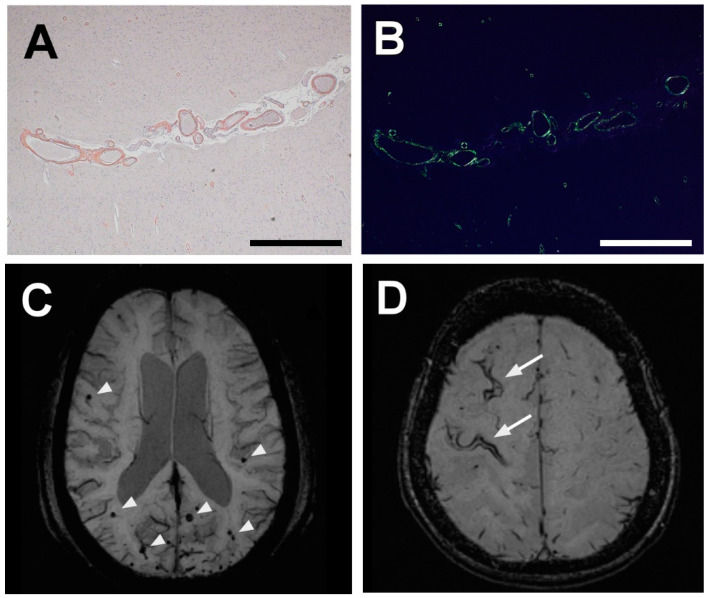
Histopathological and radiological imaging of CAA (cerebral amyloid angiopathy). Representative histopathological images from a patient with severe CAA. Congo red-positive leptomeningeal arteries (**A**) and those same arteries viewed under polarized light (**B**). SWI (susceptibility-weighted imaging) MRI shows a strictly lobar distribution of lobar microbleeds (arrowheads) (**C**). SWI shows cSS (cortical superficial siderosis) as hypointense curvilinear signals along the cortical gyri (arrows) (**D**). Scale bars = 1 mm.

With regard to pathology, the finding of apple-green birefringence under polarized light after Congo red staining has been widely used to diagnose amyloidosis, including CAA (Figure 1A,B). Aβ_40_ and Aβ_42_ are the major forms of Aβ peptides in the brain. Although Aβ_42_ differs from Aβ_40_ by only two hydrophobic residues, Aβ_42_ is more prone to aggregate compared with Aβ_40_, leading to the formation of fibrils more easily [9]. The senile plaque found in Alzheimer’s disease (AD) is predominantly composed of Aβ_42_, whereas the vascular amyloid in CAA exists as Aβ_40_ in the walls of leptomeningeal and cortical arteries [9,10]. Vascular Aβ deposits begin at the outer basement membrane of smooth muscle cells and develop into circumferential transmural Aβ deposits, thereby leading to degeneration of smooth muscle cells of the media and thickening of the vessel walls [11]. During advanced stages of CAA, Aβ disrupts the vascular extracellular matrix (ECM), which results in the “double-barrel” appearance because of the detachment and delamination of the intima from the media, microaneurysms, and fibrinoid necrosis [12].

## 3. Diagnosis of CAA: Markers, Characteristic Features, and Techniques

Advances in neuroimaging have enabled us to establish a clinical diagnosis of CAA without requiring a brain biopsy. Recently, the Edinburgh criteria for CAA-related ICH were published. In addition to including the well-known lobar location of CAA-related ICH, these criteria included a subarachnoid space extension of ICH and finger-like projections with an irregular hematoma shape, as well as the presence of the apolipoprotein E (ApoE) ε4 genotype *APOE*-ε4 [13]. 

Cerebral microbleeds (CMBs) are a diagnostic marker of the modified Boston criteria (Table 1) [14]. CMBs reflect focal hemosiderin deposits caused by previous subtle hemorrhages from small vessels and suggest the potential role of lobar CMBs as markers of CAA [15,16] (Figure 1C). A community-based study demonstrated that the prevalence of lobar CMBs was 19% among healthy elderly subjects, and a strictly lobar distribution was found in 58.4% of those cases [17]. Such a strictly lobar CMB distribution was strongly predictive of CAA in a memory clinic-based cohort, with a positive predictive value of 87.5%, which indicated that lobar CMBs may be a sensitive CAA marker in patients with cognitive impairment [18].

Cortical superficial siderosis (cSS) is another important diagnostic marker included in the modified Boston criteria (Figure 1D) [14]. cSS is thought to result from repeated leakage of blood from fragile CAA-affected vessels into the subarachnoid space; it manifests as hemosiderin deposits in the cortical sulcus. Blood-sensitive magnetic resonance imaging (MRI) sequences, including T2*-weighted gradient-echo and susceptibility-weighted imaging (SWI) sequences, demonstrate cSS as track-like curvilinear hypointense lesions on the cortical surface. The prevalence of cSS was 0.4% in population-based studies and was 2.7–3.5% in memory clinic settings [19,20]. We previously analyzed the association between cSS and the topographic distribution of CMBs at our memory clinic by using MRI (3T SWI), and we found that cSS was associated with a strictly lobar CMB location, which indicated a close relationship between CAA and cSS [20]. A meta-analysis showed that the ICH incidence per year was 11.1% in the presence of cSS and 3.9% without cSS [21]. In a CAA cohort, disseminated cSS was more frequently observed in patients with ICH than in patients without ICH (33.3% vs. 5.9%) and was associated with the *APOE*-ε2 genotype [22]. In clinical settings, cSS has been associated with transient focal neurological episodes (TFNEs), an increased risk of recurrent ICH, and new-onset dementia after ICH [23,24]. TFNEs are short stereotyped episodes of somatosensory or motor disturbance, dysphasia, and visual loss, and symptoms easily migrate from one body part to another. TFNEs seem to be triggered by cSS, and one plausible mechanism of TFNEs may be cortical spreading depression. Fifty percent of patients with TFNEs had symptomatic lobar ICHs over a median period of 14 months [25]. Therefore, recognition of TFNEs and differentiation from transient ischemic attacks are essential in clinical practice, because prescription of antithrombotics may lead to fatal ICHs. Other non-hemorrhagic markers, such as MRI-visible perivascular spaces at the centrum semiovale, are associated with CAA [26] and with the number of lobar microbleeds and cSS [27]. The centrum semiovale may reflect the failure of perivascular interstitial fluid drainage caused by vascular Aß deposits, described in the next section, thus indicating impairment in the Aß clearance system [26].

White matter hyperintensity (WMH) is also a common radiological finding in CAA. WMH manifests as bilateral and symmetrical high-intensity lesions on T2-weighted images or fluid-attenuated inversion recovery images [28]. Subcortical WMH [29] and posterior predominant WMH are frequently observed in CAA [30,31]. The pathophysiology of WMH in CAA is related to hypoperfusion associated with Aβ deposits in cortical small vessels, blood–brain barrier (BBB) disruption, loss of integrity of the cerebrovasculature, and capillary leakage from the increase in vascular permeability [1,32,33]. Increased WMH predicts a higher risk of recurrent lobar ICH in CAA, a larger hematoma volume, and hematoma enlargement [32,34].

CAA-related inflammation (CAA-ri) manifests as reversible encephalopathy and has a favorable response to immunosuppressant treatment [35]. Clinically, CAA-ri presents with progressive cognitive impairment, headache, seizures, and decreased consciousness [36,37]. Pathological analysis has revealed perivascular, intramural, and/or transmural inflammatory changes, with or without granuloma formation. Typical CAA-ri MRI images show asymmetric WMH distributed across all lobes and without preferential locations and multiple CMBs [35]. These radiological manifestations were incorporated into the diagnostic criteria of CAA-ri [36]. 

Cerebral microinfarcts are acute or subacute ischemic infarctions observed in patients with advanced CAA [38], in whom occlusive arteriopathy may be related to ischemic changes [1]. Cerebral microinfarcts manifest as small ovoid or round high intensity areas in the subcortex and cortex on diffusion-weighted MRI [38]. Analyses of autopsied brains with CAA cases have identified diffusion-weighted imaging-positive lesions that represent acute infarcts [39]. 

A previous study demonstrated that CAA is associated with thinner cortices in patients with sporadic CAA compared with healthy controls. Cortical atrophy was also observed, especially in the occipital, temporal, posterior parietal, and medial frontal regions [40].

Amyloid positron emission tomography (PET) with a radiotracer (^11^C-labeled Pittsburgh compound B (PiB) or ^18^F-florbetapir) has been reported as a promising imaging method to detect Aβ deposits [41]. Another study demonstrated that ICH or CMBs occurred at locations with greater PiB retention, which suggests the occurrence of increased vascular Aβ deposits and fragile vessels [42]. These radiotracers bind to both the vessels and the brain parenchyma [43], so differentiating vascular Aβ deposits in CAA from senile plaques in AD in PET images requires additional research. 

As a fluid matrix with potential biomarkers, cerebrospinal fluid (CSF) may be useful in the diagnosis of CAA. Reduced levels of both Aβ_40_ and Aβ_42_ in the CSF were observed in patients with CAA [44,45]. Reduced levels of Aβ_40_ and Aβ_42_ in the CSF were also found in patients with cSS [46]. Amyloid PET with the radiotracer PiB was shown to reveal vascular Aβ and to correlate with a reduced Aβ_40_ level in the CSF [47], which suggests that the CSF is a promising tool for early diagnosis and monitoring of disease progression even in the preclinical phase of CAA.

## 4. Management of CAA, Af, and ICH in Clinical Settings

Several risk factors for CAA were suggested to promote CAA in animal studies. A bilateral common carotid artery stenosis model was utilized to induce chronic cerebral hypoperfusion, which promoted CAA pathology in CAA mouse models [48]. The induction of hypertension by administering angiotensin II also resulted in enhancement of CAA [49]. With regard to diabetes mellitus, chronic diabetes generated by cross-breeding with genetically obese *ob/ob* mice worsened CAA pathology [50]. Lifestyle modifications may thus delay the onset of CAA or prevent the progression of this disease. 

A clinical dilemma exists with regard to the use of anticoagulants in patients with CAA who have atrial fibrillation (Af). The standard approach for patients with CAA who have a previous history of lobar ICH is to avoid antithrombotic therapy [51], which increases the relative risk of ICH and outweighs the reduced risk of thrombotic events. Although indications exist for the use of anticoagulants in patients with Af, the use of these agents to prevent CAA-related ICH seems to be more harmful than productive of good outcomes. A meta-analysis that included 5306 patients with anticoagulation-associated ICH demonstrated that restarting anticoagulation lowered the risk of thromboembolic events and did not increase the risk of recurrent ICH [52]. Other studies showed that restarting anticoagulants in patients with a previous ICH and Af reduced overall mortality [53,54]. However, these retrospective studies included patients with both CAA-related ICH and non-CAA-related ICH. Therefore, applying these results to clinical practice is difficult. Using anticoagulants for patients with CAA and a history of CAA-related ICH is not currently indicated [55]. Patients who had TFNE or cSS have also been treated similarly to patients with CAA-related ICH [56]. Alternative preventive approaches that are in current use include administration of direct oral anticoagulant (DOAC) and utilization of a device to occlude the left atrial appendage; these methods may be effective for patients with CAA and Af and a history of CAA-related ICH [55]. Prospective randomized trials of DOAC use in patients with CAA are warranted to answer this important question. 

Once ICH occurs, the decision to choose conservative treatments (i.e., anti-edema and anti-hypertensive treatments) versus surgical treatment is a matter of debate. Retrospective data have shown that surgical clot evacuation in patients with CAA-related ICH results in a poor outcome according to the following factors: dementia, age older than 75 years, hematoma volume and location, preoperative Glasgow Coma Scale Score 8 or lower, and intraventricular extension of the hemorrhage [57]. Here, too, prospective randomized clinical trials are needed to identify suitable treatments of CAA-related ICH.

## 5. Promising Therapeutic Targets for CAA

Although effective prevention of CAA-related ICHs or cognitive impairment is currently unavailable, several promising approaches may aid Aβ clearance and lead to CAA treatment. Aβ clearance depends mainly on three mechanisms: (1) Aβ-degrading enzymes, (2) vascular receptor-mediated clearance, and (3) the perivascular drainage pathway. First, enzymatic degradation of Aβ such as by neprilysin and insulin-degrading enzyme (IDE) plays an important role in Aβ clearance and reduces Aβ cytotoxicity. Neprilysin, identified as an Aβ-degrading enzyme, is a neural endopeptidase that cleaves Aβ in a proteolytic manner [58,59]. A recent study found a significant decrease in neprilysin levels in microvessel-enriched extracts from patients with AD [60]. Therefore, and in view of neprilysin’s reduction of brain Aβ levels in mice [61], genetic therapy to enhance neprilysin expression in the brain vasculature may be useful in CAA treatment. Another known Aβ-degrading enzyme, IDE, is also an endopeptidase that is predominantly expressed in the brain [62,63]. In addition to its insulin-degrading effects, IDE degrades Aβ and promotes Aβ removal, as in vivo studies found [64,65]. Furthermore, an in vitro study found that IDE was the major protease involved in Aβ clearance in human hippocampal lysates [66].

We recently determined that memantine, a known NMDA receptor (NMDAR) antagonist, was a promising therapy for CAA via reducing the numbers of vessels with Aβ deposits and the numbers of hemosiderin deposits in the APP23 transgenic mouse model of CAA [67]. We also found that APP23 mice manifested reduced cerebrovascular expression of IDE compared with wild-type mice and that memantine increased cerebrovascular IDE expression in APP23 mice. Past studies determined that activation of the NMDAR by treating neuronal cell cultures with NMDA led to significantly reduced IDE levels and that an NMDAR antagonist (MK801) increased IDE expression [68]. We hypothesized that reduced IDE levels are mediated by activation of the NMDAR by Aβ and, conversely, that increased IDE expression may be associated with blockage of the NMDAR by memantine. As for Aβ clearance in pathogenesis of AD, involvement of the ubiquitin-proteasome system (UPS) through its proteolytic activity was reported [69]. Further studies were warranted to identify the role of UPS in pathogenesis of CAA.

Second, with regard to the vascular receptor-mediated clearance pathway, emerging in vitro and in vivo evidence demonstrates that low-density lipoprotein receptor-related protein-1 (LRP1) is associated with clearance of Aβ from the brain. LRP1 is highly expressed in cerebrovascular walls and mediates the efflux of Aβ into the bloodstream. Kanekiyo et al. demonstrated that conditional knockout of LRP1 in vascular smooth muscle cells in APP/PS1 mice increased cerebrovascular Aβ deposits, and conditional knockout of LRP1 in neuronal cells in the brains of APP/PS1 mice resulted in impaired interstitial Aβ clearance in the cortex without affecting the Aβ-processing pathway [70,71]. Diet-based treatment with oleocanthal, a phenolic secoiridoid component of extra virgin olive oil, demonstrated enhanced LRP1 expression at the BBB, which led to improved Aβ clearance in mice [72,73].

The third mechanism, the perivascular drainage pathway, was thought to be an Aβ clearance route, and cerebrovascular Aβ deposits were believed to result from the failure of clearance of Aβ from the brain [74,75]. This mechanism was initially associated with anti-Aβ immunotherapy, which failed to demonstrate clinical efficacy in patients with AD. The worsening of CAA was believed to occur because of Aβ overload in perivascular drainage pathways via Aβ efflux from the brain caused by immunotherapy [76]. This route was described as an intramural periarterial drainage (IPAD) pathway, in which neuronal Aβ enters the perivascular drainage pathway along the basement membrane of the vascular smooth muscle cells and finally drains into the cervical lymph nodes [77,78,79]. Past ultrastructural studies of CAA have demonstrated that deposits of Aβ fibrils first appeared in the basement membrane of cerebral blood vessels [11], which indicated the failure of the IPAD pathway, because the patterns of the Aβ deposits were consistent with the IPAD pathway [80]. Furthermore, perivascular drainage of interstitial fluid depends on vessel pulsations as the motive force, and age-related functional decline (e.g., stiffening of the arteries) was hypothesized to reduce drainage flow. Certain studies proposed that vasoactive drugs promoted Aβ clearance by means of perivascular drainage-mediated Aβ clearance as a result of the motive force generated by arterial pulsations [81,82]. 

An alternative perivascular drainage pathway has been proposed in which the CSF enters the paravascular spaces and penetrates the arterial walls in the brain, mixes with interstitial fluid and solutes in the parenchyma, and removes waste products by transporting them into the paravascular spaces of the draining veins via CSF bulk flow [79]. This pathway was named the glymphatic drainage pathway, and the water channel aquaporin-4 located in the foot processes of astrocytes regulates the convective flow of fluid in the pathway [83,84]. Animal studies showed that genetic deletion of aquaporin-4 reduced the clearance of tracer and the levels of aquaporin-4 in astrocytic endfeet in CAA, which indicates the impairment of glymphatic clearance and subsequent reduction in Aβ clearance [85,86]. Although independent pathways of IPAD and glymphatic drainage coexist in relation to CSF movements, additional studies are needed to investigate which pathway is more relevant in the pathogenesis of CAA.

With regard to promising candidate therapeutics for CAA, cilostazol, a known selective inhibitor of type 3 phosphodiesterase, reduced Aβ_40_ deposits and improved cognitive impairment in CAA model mice. The distance that fluorescent Aβ tracers moved during a certain time period was significantly greater in the cilostazol-treated group than in controls, which indicated that vasoactive drugs could promote Aβ clearance [87]. Another promising therapeutic agent, taxifolin, was suggested to block Aβ oligomer formation, maintain vascular integrity, and prevent cognitive impairment in CAA model mice [88]. With regard to CAA-related ICHs, minocycline treatment reduced the numbers of microhemorrhages in the brains of CAA transgenic mice [89]. Drug repositioning to identify novel treatment indications from old drugs might be a promising strategy to benefit CAA patients. 

With respect to prevention, a sub-analysis of the PROGRESS study demonstrated that lowering blood pressure with perindopril reduced the recurrence of CAA-related ICHs by 77% during a follow-up period of 3.9 years, which supports the efficacy of strict blood pressure control to manage CAA [90].

## 6. Key Molecules in CAA Identified by Means of Proteomic Analyses

Although developing a therapeutic strategy to ameliorate CAA is urgently needed, molecules or physiological processes involved in the pathogenesis of CAA remain to be elucidated. Recently, proteomic analysis of human brain samples has garnered increasing interest in CAA as a means to identify key molecules that may lead to treatment, and upregulated molecules have been identified as novel potential therapeutic targets for CAA. We used laser capture microdissection to isolate human vascular material from CAA-affected leptomeningeal and cortical arteries from formalin-fixed paraffin-embedded (FFPE) samples obtained at autopsy [91]. Quantitative liquid chromatography-tandem mass spectrometry demonstrated upregulation of ApoE, clusterin, and sushi repeat-containing protein X-linked 1 (SRPX1) in CAA-affected vessels. 

Of these upregulated molecules, we focused on SRPX1 and undertook a neuropathological study. SRPX1, a transmembrane protein consisting of 464 amino acids, has three sushi domains, which are found in various complement and adhesion proteins; it also has a short intracellular domain in the C-terminal region [92,93,94]. *SRPX1* was first identified as a causative gene in patients with X-linked retinitis pigmentosa [95]. SRPX1 mRNA expression was downregulated in tumor cells, and the gene was believed to be a tumor suppressor gene [93,94,96]. We found that SRPX1 co-accumulated with vascular Aβ deposits, and its occurrence increased with CAA severity. SRPX1 mRNA and protein levels also increased in primary cultures of cerebrovascular smooth muscle cells treated with Aβ_40_. In addition, we discovered that SRPX1 bound to Aβ in vitro and enhanced Aβ-induced caspase activities in cultured cells, which led to Aβ-induced cerebrovascular degeneration in CAA. 

Endo et al. performed proteomic analyses of microdissected specimens of leptomeningeal and cortical vessels from surgical FFPE samples of lobar ICHs, and they identified ApoE and clusterin as upregulated molecules and investigated their roles in relation to Aβ aggregation [97]. They also used a unique in vitro CAA model of the IPAD pathway and found that ApoE and clusterin delayed the start of amyloid fibril formation [97,98].

Manousopoulou et al. used human leptomeningeal arteries isolated from frozen autopsy samples of severe capillary CAA and detected tissue inhibitor of metalloproteinases 3 (TIMP3) and clusterin as upregulated proteins compared with non-CAA age-matched controls [99]. Hondius et al. used microdissected human gray matter samples from frozen autopsy samples of CAA-affected capillaries and compared these samples with those without CAA. They identified HTRA1 (HtrA serine peptidase 1) as a CAA-specific molecule that accumulated in the vessels. They observed HTRA1 in all the CAA cases, only one AD case, and no control case [100]. Although each study utilized different target blood vessels in CAA and different specimen types (FFPE or frozen brain samples), data from all these studies taken together may provide resources for generating novel therapeutic targets for CAA. Of the upregulated molecules described above, ApoE and clusterin were commonly noted. *APOE* is the confirmed susceptibility locus for CAA identified in genetic analyses [101,102]. Proposed mechanisms of ApoE isoform-specific effects are direct competition for Aβ clearance [103] and effects on peptide aggregation or fibrillogenesis [104]. Of the three *APOE* genotypes (ε2, ε3, and ε4), the *APOE*-ε4 genotype is a major risk factor for both AD [105] and CAA [106], and *ApoE*-ε2/ε4 genotype carriers are prone to develop CAA at an early age [107]. CAA is classified into two subtypes: the first is CAA type 1, or capillary CAA, which is characterized by Aβ deposits in the capillaries regardless of the involvement of larger vessels. The second, CAA type 2, is characterized by Aβ deposits in the leptomeningeal and cortical arteries [108,109]. CAA type 1 leads to capillary occlusion and impaired vascular perfusion and then to dementia [110]. CAA type 1 is also associated with the *APOE*-ɛ4 genotype [108,111], whereas the *APOE*-ε2 genotype is associated with CAA type 2 [111,112]. Strictly lobar CMBs are also associated with the *APOE*-ε4 genotype [17,113,114], whereas the *APOE*-ε2 genotype is associated with cSS [22], CAA-related ICH [106], and larger hematoma volume [115]. Inasmuch as the *APOE*-ε2 genotype is also associated with fiprobrinoid necrosis in CAA [116,117], possession of the *APOE*-ε2 genotype may be related to the loss of structural integrity of the cerebrovasculature.

Immunohistochemical studies demonstrated co-localization of Aβ and ApoE in the perivascular spaces, which indicated the involvement of ApoE in the perivascular drainage pathway. Although in vitro analyses showed inconsistent effects of ApoE on Aβ fibril formation [118,119], a study with the CAA/AD mouse model that had a genetic deletion of murine ApoE revealed few vascular and parenchymal Aβ deposits, which indicated enhancement of Aβ plaque formation by ApoE [120]. ApoE-dependent reduction of Aβ deposits was also seen in the CAA/AD mouse expressing human ApoE [121]. 

Clusterin, also known as apolipoprotein J, is a ubiquitously expressed protein present in a wide range of tissues [122]. It is a 70–80kDa highly glycosylated protein that is cleaved to form α- and β-subunits linked together by disulfide bonds [123]. Clusterin is involved in a number of cellular processes as an extracellular chaperone, as well as in cell survival and cell death pathways [124]. Clusterin also binds to Aβ oligomers and inhibits Aβ aggregation and amyloid fibril formation [125]. In addition, clusterin mediates its Aβ clearance across the BBB via lipoprotein-related protein-2 (LRP2, known as megalin) [126,127]. A recent study demonstrated worsened CAA in clusterin-knockout mice, and the loss of clusterin likely reduced Aβ clearance across the BBB and shifted the clearance pathway toward perivascular drainage routes [128]. 

TIMP3 is a 25 kDa protein containing disulfide bonds that is found in the central nervous system [129]; it is also an endogenous tissue inhibitor involved in ECM homeostasis by inhibiting matrix metallopeptidase 9 activity [130,131]. Genetic deletion of TIMP3 resulted in pathological arterial vasodilation in mice [131]. The results in that study showed that expression of TIMP3 was restricted to the walls of leptomeningeal arteries, which indicated a role of TIMP3 in preserving cerebrovascular integrity by ensuring a balance with the activities of matrix metalloproteinases [99].

HTRA1, a member of the serine protease family, cleaved ECM proteins such as collagen and fibronectin [132]. HTRA1 also degraded Aβ in vitro and co-localized with parenchymal and perivascular Aβ deposits [133]. In addition, mutations in HTRA1 were identified as a cause of the hereditary small vessel disease CARASIL (cerebral autosomal recessive arteriopathy with subcortical infarcts and leukoencephalopathy) [134]. Upregulated TIMP3 or HTRA1 levels in CAA-affected blood vessels may reflect vascular ECM remodeling as a compensatory reaction to vascular Aβ accumulation.

## 7. Future Perspectives

CAA presents in two ways: neurodegenerative disease and cerebrovascular disease, and Aβ deposits in vessel walls can result in ICH, ischemic stroke, and dementia. However, the molecular pathways that directly influence the progression of CAA have not yet been clarified. The molecules that have been identified by the proteomic analyses described above may be key target molecules in CAA and may be applied in future studies to determine the involvement of those molecules in the pathogenesis of CAA. Chemical compounds or genetic therapy to enhance expression of target molecules in brain vasculature may be effective in CAA treatment. Furthermore, determination of the chronological alterations in molecules throughout the progression of this disease is important to understand the pathogenesis of CAA. Early accurate diagnosis of CAA is quite important for the utilization of early preventive and disease-modifying treatments. Therefore, continued research to discover fluid or radiological biomarkers that allow early diagnosis of CAA will also be needed to develop those treatments. Reductions in the incidence of ICH and dementia resulting from CAA will decrease the emotional distress and physical impairment of all patients and their caregivers and will foster improved medical care around the world.

## Figures and Tables

**Table 1:** Modified Boston criteria for CAA diagnosis.

Diagnosis	Description
**Definite CAA**	Full postmortem examination demonstrating:
	▪ Lobar, cortical, or corticosubcortical hemorrhage
	▪ Severe CAA with vasculopathy
	▪ Absence of another diagnostic lesion
**Probable CAA with supporting pathology**	Clinical data and pathological tissue (evacuated hematoma or cortical biopsy) demonstrating:
	▪ Lobar, cortical, or corticosubcortical hemorrhage
	▪ Some degree of CAA in specimen
	▪ Absence of another diagnostic lesion
**Probable CAA**	Clinical data and MRI or CT demonstrating:
	▪ Multiple hemorrhages restricted to lobar, cortical, or corticosubcortical regions (cerebellar hemorrhage allowed) *OR* A single lobar, cortical, or corticosubcortical hemorrhage and focal or disseminated * superficial siderosis
	▪ Age ≥ 55 years
	▪ Absence of other cause of hemorrhage or superficial siderosis
**Possible CAA**	Clinical data and MRI or CT demonstrating:
	▪ Single lobar, cortical, or corticosubcortical hemorrhage *OR* Focal or disseminated * superficial siderosis
	▪ Age ≥ 55 years
	▪ Absence of other cause of hemorrhage or superficial siderosis

* Focal superficial siderosis: siderosis restricted to three or fewer sulci; disseminated superficial siderosis: siderosis affecting at least four sulci. CT, computed tomography.

## Data Availability

Not applicable.

## Abbreviations

CAA	Cerebral amyloid angiopathy
ICH	Intracerebral hemorrhage
AD	Alzheimer’s disease
Aβ	Amyloid β
TFNEs	Transient focal neurological events
WMH	White matter hyperintensity
CAA-ri	CAA-related inflammation
PiB	^11^C-labeled Pittsburgh compound B
PET	Positron emission tomography
AF	Atrial fibrillation
IPAD	Intramural periarterial drainage
CSF	Cerebrospinal fluid
SRPX1	Sushi repeat-containing protein X-linked 1
TIMP3	Tissue inhibitor of metalloproteinases 3
HTRA1	HtrA serine peptidase 1
LRP-1	Low-density lipoprotein receptor-related protein 1
ApoE	Apolipoprotein E
IDE	Insulin-degrading enzyme
BBB	Blood-brain barrier
ECM	Extracellular matrix
CMBs	Cerebral microbleeds
cSS	Cortical superficial siderosis
MRI	Magnetic resonance imaging
SWI	Susceptibility-weighted imaging
NMDAR	NMDA receptor
UPS	Ubiquitin-proteasome system
